# Branch Chain Amino Acid Metabolism Promotes Brain Metastasis of NSCLC through EMT Occurrence by Regulating ALKBH5 activity

**DOI:** 10.7150/ijbs.85672

**Published:** 2024-06-29

**Authors:** Luning Mao, Lan Wang, Yingying Lyu, Qiyuan Zhuang, Zhujun Li, Jialong Zhang, Zhiyan Gu, Shaohua Lu, Xin Wang, Yun Guan, Ji Xiong, Yin Wang, Ying Mao, Hui Yang, Ying Liu

**Affiliations:** 1 Department of Pathology, School of Basic Medical Sciences, Huashan Hospital, Fudan University; 2 Department of Neurosurgery, Huashan Hospital, Fudan University.; 3 Department of Pathology, Zhongshan Hospital, Shanghai Medical College, Fudan University.; 4 Cyberknife Centre, Department of Neurosurgery, Huashan Hospital, Fudan University; 5 Department of Pathology, Huashan Hospital, Shanghai Medical College, Fudan University.; 6 National Centre for Neurological Disorders, Huashan Hospital, Institute for Translational Brain Research, State Key Laboratory of Medical Neurobiology and MOE Frontiers Centremete for Brain Science, Fudan University, Shanghai.

**Keywords:** NSCLC, BCAT1, α-KG, ALKBH5, EMT

## Abstract

Metabolic reprogramming is one of the essential features of tumors that may dramatically contribute to cancer metastasis. Employing liquid chromatography-tandem mass spectrometry-based metabolomics, we analyzed the metabolic profile from 12 pairwise serum samples of NSCLC brain metastasis patients before and after CyberKnife Stereotactic Radiotherapy. We evaluated the histopathological architecture of 144 surgically resected NSCLC brain metastases. Differential metabolites were screened and conducted for functional clustering and annotation. Metabolomic profiling identified a pathway that was enriched in the metabolism of branched-chain amino acids (BCAAs). Pathologically, adenocarcinoma with a solid growth pattern has a higher propensity for brain metastasis. Patients with high BCAT1 protein levels in lung adenocarcinoma tissues were associated with a poor prognosis. We found that brain NSCLC cells had elevated catabolism of BCAAs, which led to a depletion of α-KG. This depletion, in turn, reduced the expression and activity of the m6A demethylase ALKBH5. Thus, ALKBH5 inhibition participated in maintaining the m6A methylation of mesenchymal genes and promoted the occurrence of epithelial-mesenchymal transition (EMT) in NSCLC cells and the proliferation of NSCLC cells in the brain. BCAA catabolism plays an essential role in the metastasis of NSCLC cells.

## Background

Lung cancer is the leading cause of cancer-related mortality 1. Non-small cell lung carcinoma (NSCLC) accounts for approximately 80% of all lung cancer cases and can be divided into three main categories: squamous cell carcinoma, adenocarcinoma, and large cell carcinoma 2. Metastasis is one of the leading causes of death in patients with lung cancer 3. The brain is the most common site of lung cancer metastasis, with up to 50% of lung cancers presenting with brain metastases (BMs) in advanced stages 4,5. The incidence of BM is high in NSCLC compared to other common epithelial malignancies 6. NSCLC is predisposed to BM for unknown reasons.

As with normal cells, tumor cells use glucose, amino acids, and other nutrients to support their growth. Recent studies have shown that tumor cells have a different metabolism than normal cells. Reprogramming of the metabolism is an essential characteristic of cancer cells 7. Metabolic reprogramming in cancer cells reshapes the abundance of various metabolites. On the one hand, cancer cells can perceive and utilize specific metabolites to maintain the nutrients required for malignant tumor proliferation 8. On the other hand, some metabolites can be used by cancer cells to regulate epigenetic and epitranscriptomic modifications, affecting genome integrity, and function as intracellular signaling molecules 9,10. In the process of tumor cell metastasis, tumors selectively and dynamically reprogram specific metabolic pathways to promote the formation and growth of tumor metastases 11.

An elevated demand for amino acids supports rapid cell proliferation and cancer progression 12. Branched-chain amino acids (BCAAs), including L-valine, L-leucine, and L-isoleucine, are essential amino acids. Branched-chain amino acid aminotransferase (BCAT) is a key enzyme that catalyzes the metabolism of BCAAs and has two isozyme subtypes. BCAT1 is located in the cytoplasm, while BCAT2 is mainly present in mitochondria 13. BCAAs can be swiftly transported into the brain and act as a nutrient source for cells in the brain 14. Metabolic reprogramming of BCAAs is closely associated with the increased proliferation capacity of various tumor cells 15. However, whether it is involved in the invasive growth of brain metastatic lung cancer cells is still unknown.

In this study, metabolomics profiling revealed distinct alterations in NSCLC brain metastasis before and after CyberKnife Stereotactic Radiotherapy. Differential metabolites were screened and conducted for functional clustering and annotation. A significantly enriched pathway was found involving BCAAs metabolism. We analyzed the pathology subtypes of 144 brain metastases and compared the growth patterns of 118 brain metastases from lung adenocarcinoma with those of 72 lung adenocarcinoma cases. We found that solid and micropapillary proliferation patterns were the main growth patterns of lung adenocarcinoma brain metastases. BCAT1 was highly expressed in lung adenocarcinoma brain metastases. Furthermore, using LC-MS/MS, we confirmed that BCAT1 catalyzes BCAAs to branched-chain α-keto acids (BCKAs) in NSCLC cells and that α-KG levels in cells were significantly increased after the abolishment of catabolism. The levels of α-KG modulated the expression and activity of ALKBH5, an α-KG-dependent m6A demethylase, which further manipulated the mRNA methylation levels of EMT genes and was involved in the malignant proliferation of NSCLC cells in the brain.

## Materials and methods

### Patients

Patients who had been diagnosed as having NSCLC brain metastasis by histopathological confirmation before CyberKnife Stereotactic Radiotherapy at Huashan Hospital affiliated to Fudan University between June 1, 2020, and October 31, 2021, and who met the following criteria were enrolled in the study: (1) participants were not suffering from other metabolic, liver, or kidney diseases or any other tumors; (2) preoperative blood samples after overnight fasting were collected after withdrawal of any medication; and (3) postoperative blood samples after overnight fasting were collected within 1 to 3 days after successful CyberKnife Stereotactic Radiotherapy.

This study was approved by the Local Ethics Committee of Fudan University. Informed consent was obtained from all of the participants before entering the study. The baseline characteristics of enrolled NSCLC BM patients are described in supplementary Table 1S. This study included 6 male and 6 female patients, with a mean age of 65.7±10.16 years and a median disease duration of 21 months.

### Serum Collection and Preparation for UPLC-MS/MS

Fasting whole blood samples were collected in a Vacutainer tube. The blood samples were kept at room temperature for 30 min for clotting. Clotted blood samples were centrifuged at 3000 × g at 4°C for 20 min to remove the supernatant serum and quickly stored at -80°C until assayed.

Serum (100 μL) was added into 400 μL methanol (pre-chilled to -20 °C) to make a final 80% (v/v) methanol solution. After incubation at -20 °C for 2 to 4 hours, all sample solutions were centrifuged at 12000 × g for 20 minutes. All supernatants were dried and re-dissolved in 100 μL of 80% (v/v) methanol solution for UPLC-MS/MS analysis. A pooled quality control (QC) sample was prepared by mixing 5 μL of extracted solution from each sample into autosampler vial. Pooled samples were analyzed every twenty injections during the LC-MS analysis to monitor the stability of the data acquisition and used for subsequent data normalization.

Both HILIC-MS and RPLC-MS approaches were used for comprehensive analysis of the metabolome. A Welch Ultimate AQ-C18 column (particle size, 5μm) and SeQuant ZIC-HILIC column (particle size, 5μm) were used for separation of metabolites. Thermo Q Exactive HF (Xcalibur, Thermo Scientific, San Jose, CA, USA) was operated in full MS-scan mode for data acquisition

### LC-MS data processing

The resulting mass spectra were exported into Compound Discoverer 3.1 (Thermo Scientific, San Jose, CA, USA) for further processing. A predefined untargeted workflow named “Untargeted Metabolomics with statistics detect unknowns with ID using Online Database and mzLogic” was used. mzCloud was used to annotate compounds on MS/MS level with a mass tolerance of 10 ppm. Chemspider, which integrates BioCyc, Human Metabolome Database, and KEGG database, was used to annotate features based on exact mass with a mass tolerance of 5 ppm as well as the CD internal database.

### NSCLC samples

A primary lung adenocarcinoma tissue microarray (luc1601, 0.6-mm tissue cores) was commercially obtained from Shanghai Zhuohao Pharmaceutical Co., Ltd. (Shanghai, China). Patient ages ranged from 30 to 74 years (median = 60 y), and the pathologic stages ranged from Ia to IV. A total of 144 NSCLC brain metastases were collected from patients that underwent neurosurgical resection in the Department of Neurosurgery of Huashan Hospital affiliated with Fudan University from January 2013 to December 2016. The clinical data of the patients were collected, including sex, age, tumor metastasis site, and other information. All the tested cases were approved following the ethics of Fudan University, and the consent of the patients was obtained. This study was approved by the Institutional Review Board of Fudan University (Y2021-Y022) and was performed in accordance with the Declaration of Helsinki (1964, amended most recently in 2013) and the ethical principles of the World Medical Association 16.

### Histopathologic evaluation

The above paraffin specimens were stained with hematoxylin & eosin (HE). The poorly differentiated samples were distinguished by necessary immune markers, including P40 (Maixin, RMA0818), TTF1 (DAKO, M3575), Napsin A (Leica, 6060530), CD56 (DAKO, M7304), Chromogranin A (DAKO, M0869) and Synaptophysin (DAKO, M7315). Immunostaining for TTF1, Napsin A, and P40 was used to differentiate adenocarcinoma (TTF1 positive, Napsin A positive, P40 negative) and squamous carcinoma (TTF1 negative, Napsin negative, P40 positive). Staining for synaptophysin, Chromogranin A, and CD56 was used to exclude neuroendocrine carcinoma. Two pathologists (Y.L., SH. L.) jointly confirmed the histopathologic subtypes of NSCLC brain metastases according to the current WHO diagnostic standard 2. The lung adenocarcinoma samples were further classified according to their architectural subtypes as acinar, papillary, solid, or micropapillary.

### Cell culture and treatment

H1299 and A549 human NSCLC cells were obtained from the American Type Culture Collection (ATCC) and cultured in Ham's F-12K medium (for H1299 cells) and RPMI 1640 medium (for A549 cells) supplemented with 10% fetal bovine serum (FBS), 100 U/mL penicillin G, and 100 mg/mL streptomycin sulfate. The culture medium, FBS, and antibiotics were from Gibco-BRL Life Technologies (Gaithersburg, MD. USA). 2-HG was added to the culture medium as described.

### Antibodies and reagents

Antibodies against human BCAT1 (rabbit, Proteintech, 19003-1-AP; dilution 1:100), ALKBH5 (16837-1-AP; dilution 1:200), and N-cadherin (4061, dilution 1:1000) were used for immunohistochemistry staining.

Antibodies against human 5-hydroxymethylcytosine (5hmC, active motif, Carlsbad, CA, dilution at 1:4000) and m6A (Abcam, diluted at 1:1000) were used for dot blotting.

For western blotting, antibodies against human BCAT1 (13640-1-AP, dilution 1:1000), ALKBH5 (16837-1-AP, dilution 1:2000), and FTO (27226-1-AP, dilution 1:1000) were commercially acquired from Proteintech Group, Inc. (Rosemont, IL, USA) and antibodies against N-cadherin (4061, dilution 1:1000), E-cadherin (3195, dilution 1:1000), and vimentin (5741, dilution 1:1000) were commercially acquired from Cell Signaling Technology, Inc. (Danvers, Massachusetts, USA).

For NSCLC diagnosis, antibodies against TTF1 (M3575), CgA (M0869), CD56 (M7304), and syn (M7315) from Agilent Technologies Co., Ltd. (Santa Clara, CA, USA), against Napsin A (6060530) from Thermo Fisher Scientific Inc. (Waltham, MA, USA), and against p40 (RAM0818) from Maixin Biotechnology Development Co., Ltd. (Fuzhou, Fujian, China) were applied for immunohistochemistry using a LEICA BOND-MAX automatic immunohistochemistry system (Thermo Fisher Scientific Inc., Waltham, MA, USA).

The metabolite standards used for LC-MS/MS, including L-valine (L-Val), L-isoleucine (L-Ile), L-leucine (L-Leu), and α-ketoglutarate (α-KG), were purchased from Sigma‒Aldrich (St Louis, MO, USA). High-performance LC grade acetonitrile, methanol, and ethanol were purchased from Tedia (Fairfield, OH, USA). The ultrapure water was produced with a Milli-Q reagent water system (Millipore, Bedford, MA, USA). 5-Sulfosalicylic acid (5-SSA) was purchased from Tokyo Chemical Industry Co. (Tokyo, Japan).

### Quantitative analysis of BCAA metabolites via LC-MS/MS

One million cells were collected and resuspended in 800 ml of ice-cold 80% methanol and 20% ddH_2_O. Samples were vigorously vortexed and placed in liquid N_2_ for 10 min to freeze and then thawed on ice for 10 min. The freeze-thaw cycle was repeated twice. Samples were centrifuged at 13,000 × g for 15 min to pellet cell debris, lipids, and proteins. The supernatant was evaporated, and the resulting metabolites were resuspended. Metabolites were normalized based on protein concentration. Standard solutions of the BCAAs, glutamate, and α-KG were prepared to a final concentration of 10 µmol/mL in 50% (v/v) methanol (1:1 methanol-water). Liquid chromatography-tandem mass spectrometry (LC-MS/MS) analysis was performed using a QTRAP3200 system (AB SCIEX) equipped with an electrospray ionization ion source and an UltiMate3000 HPLC system (Dionex/Thermo Fisher) consisting of a dual-ternary gradient pump. Data were analyzed with Analyst ver. 1.5.1 software (AB SCIEX). An N-EVAP nitrogen evaporator was acquired from Shimadzu Corporation.

### Western blot analysis

For western blot analysis, proteins were extracted from cells in radioimmunoprecipitation buffer (RIPA) containing complete protease and phosphatase inhibitor cocktails (Selleck, Houston, TX, USA), and the protein concentration was determined using a BCA Protein Assay Kit (Thermo Fisher Scientific, Waltham, MA, USA). A total of 30 μg of protein was loaded and separated in a 10% SDS‒PAGE gel and then transferred to a PVDF membrane (Millipore, MA, USA). Blots were incubated with primary antibodies followed by incubation with HRP-tagged secondary antibodies (Abcam, Cambridge, UK). The protein-antibody complexes were visualized using an enhanced chemiluminescence kit (Thermo Fisher Scientific, Waltham, MA, USA).

### shRNA Knockdown

Knockdown of BCAT1 in H1299 and A549 lung adenocarcinoma cells was achieved using lentiviral shRNA vector systems. Lentiviral shRNA vectors for human BCAT1 and the control vector were purchased from Genepharma Company (Shanghai, China). Puromycin (1 µg/ml) (Sigma‒Aldrich, Munich, Germany) was used to eliminate non-transfected cells three days after transfection The sequence of BCAT1 shRNA was GCAGGTCCTGTGGCTCTATGG.

ALKBH5 siRNA was commercially acquired from Hanbio Ltd, Shanghai, China, and the siRNA sequences were (F) 3'-CUGCGCAACAAGUACUUCUTT-5' and (R) 3'-AGAAGUACUUGUUGCGCAGTT-5'.

### Cell proliferation assay

H1299 and A549 human NSCLC cells (2×10³/well) were seeded in a 96-well plate and incubated in conditioned media with different treatments indicated in the figure legends or with the carrier control (DMSO). Cell proliferation rates were determined using Cell Counting Kit-8 (CCK-8) (Dojindo Laboratories, Japan) according to the manufacturer's instructions.

### Transwell migration assay

Transwell migration assays were performed on H1299 and A549 human NSCLC cells using a Boyden chamber containing a polycarbonate filter with an 8 μm pore size (Corning Costar, Corning, NY). The lower chambers were filled with 600μl completed endothelial culture medium (ECM, PromoCell, Heidelberg, Germany). Approximately 5×10^5^ lung adenocarcinoma cells were serum-starved for 24 h, suspended in 200 μL of serum-free ECM and added to the upper chamber. Cells were incubated at 37 °C for 12 h to allow cell migration. Migratory cells were fixed in 4% paraformaldehyde and stained with crystal violet. Images were captured and analyzed using ImageJ software (NIH, Bethesda, MD).

### Wound healing assay

Lung adenocarcinoma cells were seeded and cultured to 90% confluence in a 6-well plate before wounding with a 200 mL pipette tip. The culture medium was replaced with 2 mL of DMEM. Images were taken at 0 h and 24 h after scratching.

### Immunohistochemistry staining

Sections (4 μm thick) of formalin-fixed/paraffin-embedded tissues were deparaffinized in xylene and rehydrated in graded ethanol. Antigen retrieval was performed by heating in citrate buffer (10 mM, pH 6.0) for 10 min. Slides were incubated with primary antibodies for 1.5 h at RT. Immunoreactive elements were visualized with an EnVision Detection kit (cat# GK500705; Dako Agilent Technologies, Inc.) containing a secondary antibody and the peroxidase/3,3-diaminobenzidine (DAB) chromogen. Then, the cell nuclei were counterstained with hematoxylin. Slides not treated with primary antibody were used as a negative control.

To quantify IHC staining density, five highly positive staining areas were selected from each sample, and images were taken using a charge-coupled device camera. Images were processed and analyzed using IMT i-Solution software (IMT i-Solution, Inc., Burnaby, BC, Canada). IHC staining density was calculated as the mean ± SEM. According to the IHC staining density, the cases were divided into a high expression group with a staining intensity above the average value and a low expression group with a staining intensity lower than the average value.

### Dot-blot assays

We followed procedures described previously 17. Briefly, genomic DNA or mRNA was spotted onto nitrocellulose membranes. The membrane was baked at 80 °C and then blocked with 5% skimmed milk in TBST for 1 h, followed by incubation with an anti-5hmC antibody overnight at 4 °C and HRP-conjugated anti-rabbit IgG secondary antibody for 1 h at room temperature. After three washes with TBST, the membrane was treated with ECL and scanned with a Typhoon scanner. The quantification of dot blots was performed using Image-Quanta software (GE Healthcare).

### Quantitative real-time PCR

Total RNA was isolated from cells using TRIzol and reverse transcribed to cDNA (Invitrogen), followed by amplification via quantitative real-time PCR (qPCR) with gene-specific primers and SYBR Premix Ex-Taq (Takara). PCRs were performed in triplicate, and the relative mRNA level was calculated using the comparative CT method with Actin b as a control. The primer sequences used for qPCR were as follows:

NCAD-F 5'-CCCAAGACAAAGAGACCCAG-3'

NCAD-R 5'-GCCACTGTGCTTACTGAATTG-3'

ECAD-F 5'-CCCAATACATCTCCCTTCACAG-3'

ECAD-R 5'-CCACCTCTAAGGCCATCTTTG-3'

SNAIL-F 5'-ACAAGCACCAAGAGTCCG-3'

SNAIL-R 5'-ATGGCAGTGAGAAGGATGTG-3'

SLUG-F 5'-ACTGCTCCAAAACCTTCTCC-3'

SLUG-R 5'-TGTCATTTGGCTTCGGAGTG-3'

VIM-F 5'-GACGCCATCAACACCGAGTT-3'

VIM-R 5'-CTTTGTCGTTGGTTAGCTGGT-3'

ZEB1-F 5'-ACCCTTGAAAGTGATCCAGC-3'

ZEB1-R 5'-CATTCCATTTTCTGTCTTCCGC-3'

ACTB-F 5'-ACCTTCTACAATGAGCTGCG-3'

ACTB-R 5'-CCTGGATAGCAACGTACATGG-3'

### m^6^A immunoprecipitation qPCR (MeRIP-PCR)

The m^6^A immunoprecipitation assay was adopted from a previous protocol with several modifications 18. Briefly, purified RNA was denatured at 94 °C for 5 minutes. Fragmented RNA was incubated with m6A antibody in RIP buffer (150 mm NaCl, 10 mm TRIS-HCl, and 0.1% NP-40) supplemented with RNase inhibitor for 2 h at 4 °C. Then, Protein G beads (Invitrogen) were washed and incubated with the mixture for 2 h at 4 °C. Beads were washed three times with RIP buffer, and m6A RNA was eluted twice with m6A 5'-monophosphate sodium salt. The samples were precipitated by ethanol. The m6A enrichment was determined by real-time PCR.

GGACT site1

F: GGAAAGAGGAGCCGAGAAG

R: GCUCGUGGUUUUGCUUCCU

GGACT site2

F: CCUCCACACACUCGCAGA

R: AAUGGAGAGCGAGCUGAUGA

### Analysis of publicly available datasets

RNA-seq expression profiles and clinical characteristics of the cancer genome atlas (TCGA) lung adenocarcinoma (LUAD) cohort were downloaded. The RNAseq data from 504 LUAD samples were collected and preprocessed using the “ggpubr” R packages to analyze the expressions of ALKBH5 and FTO into upper 1/3 high and lower 1/3 BCAT1 expression groups.

### Orthotopic transplantation

Animal experiments were carried out after receiving approval from the Institutional Animal Care and Use Committee (IACUC) of Fudan University (20180302-051). BALB/c nude mice (female, 8 weeks old) were purchased from Shanghai SIPPR-BK Laboratory Animal Company (Shanghai, China) and randomly divided into two groups (BCAT1wt and shBCAT1 groups), with 16 mice in each group. Each mouse was stereotactically injected in the left striatum with a 6-µL volume (2×10^5^) of H1299 cells transfected with luciferase. The mice were monitored and euthanized when they exhibited signs of morbidity, including hunched posture, lethargy, difficulty ambulating, and weight loss, or after one month. On Day 28, all the mice were sacrificed, and the mouse brains were removed, cut conically to obtain the largest segmentation of the tumor, formalin-fixed, paraffin embedded, and cut into slides for H&E staining and immunostaining.

ImageJ software (U.S. National Institutes of Health, https://imagej.nih.gov/ij/download.html) with customized macros was used to quantify tumor areas that were defined by two neuropathologists.

### Bioluminescent Imaging

The mice were anesthetized with an intraperitoneal injection of D-luciferin potassium (150 mg/kg; Caliper Life Sciences, Xenogen Corp., Hopkinton, MA, USA). Ten minutes after luciferin injection, the mice were imaged with an IVIS 2.50 cooled charged-coupled device camera (Xenogen 200 series). For BLI analysis, an intracranial area of the signal was defined using Living Image software, and the total number of photons per second per steradian centimeter was recorded.

### Statistical analysis

Statistical analysis was performed using GraphPad Prism, version 7.0 (La Jolla, CA). Paired and unpaired t-tests or one-way ANOVA, followed by Scheffe's post hoc tests, were conducted. All experiments were performed at least three times. Progression-free survival (PFS) and overall survival (OS) curves were generated using the Kaplan-Meier method. Comparisons of the survival distribution were made with a log-rank test. The data shown represent the mean ± SEM. A value of P < 0.05 was considered significant (*P < 0.05, **P < 0.01, ***P < 0.001, and n.s. no significance).

### Study approval

All of the patients signed informed consent prior to this study. The Ethics Committee of Fudan University approved the procedures associated with the acquisition and usage of human NSCLC samples (2021-Y022). Animal experiments were carried out after receiving approval from the Institutional Animal Care and Use Committee (IACUC) of Fudan University (20210302007).

### Role of the funding source

The funders of the study had no role in study design, data collection, data analysis, data interpretation, writing of the report and decision to submit the paper for publication.

## Results

### NSCLC brain metastases-related serum metabolites

**Figure 1:**
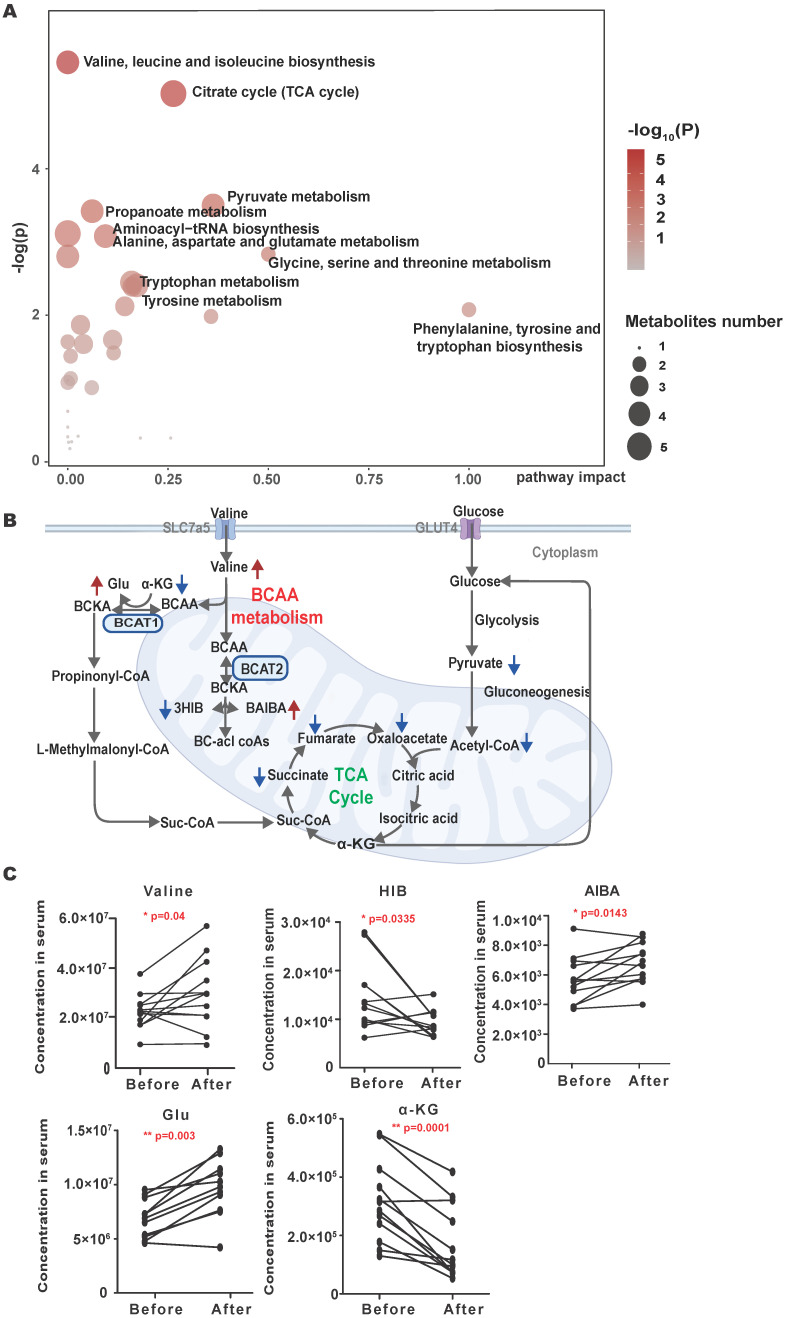
** Overall serum metabolic profile of NSCLC brain metastases patients before and after CyberKnife Stereotactic Radiotherapy. (A)** Plots depict the computed metabolic pathways as a function of -log(p) (y-axis) and the pathway impacts of the key metabolites (x-axis) that differed after CyberKnife Stereotactic Radiotherapy. **(B)** the schematic map of the mitochondrial citric acid cycle and metabolism pathways. The green arrows are metabolites that rise after treatment; the red arrows are metabolites that decrease after CyberKnife Stereotactic Radiotherapy. **(C)** The concentrations of the examined metabolites in BCAAs metabolism pathway. Significance was assessed with paired t-tests. *P<0.05. **P<0.01, *** P<0.001.

To identify metabolic changes that may be related to brain metastasis of NSCLC, a pairwise serum metabolomics comparison of NSCLC brain metastases patients before and after CyberKnife Stereotactic Radiotherapy was conducted. A total of 213 serum metabolites were identified. Thirty-six metabolites have changed before and after treatment, including an increase of 13 metabolites and a decrease of 23 metabolites. The pathway analysis showed the detailed impacts of brain metastasis of NSCLC-related alterations in metabolic networks. The most influential metabolic pathway revealed changes in the metabolism of BCAAs and the citrate cycle (Fig. 1A). In the metabolic profile, decreased levels of the mitochondrial citric acid cycle and elevated levels of BCAAs metabolism achieved higher significance (Fig.1B). The concentrations of valine were significantly increased after the radiotherapy of brain metastasis of NSCLC (Fig. 1C), and the changes of metabolites of the citric acid cycle are shown in Supplementary Fig. 1S.

### Pathological analysis of NSCLC brain metastases

**Figure 2:**
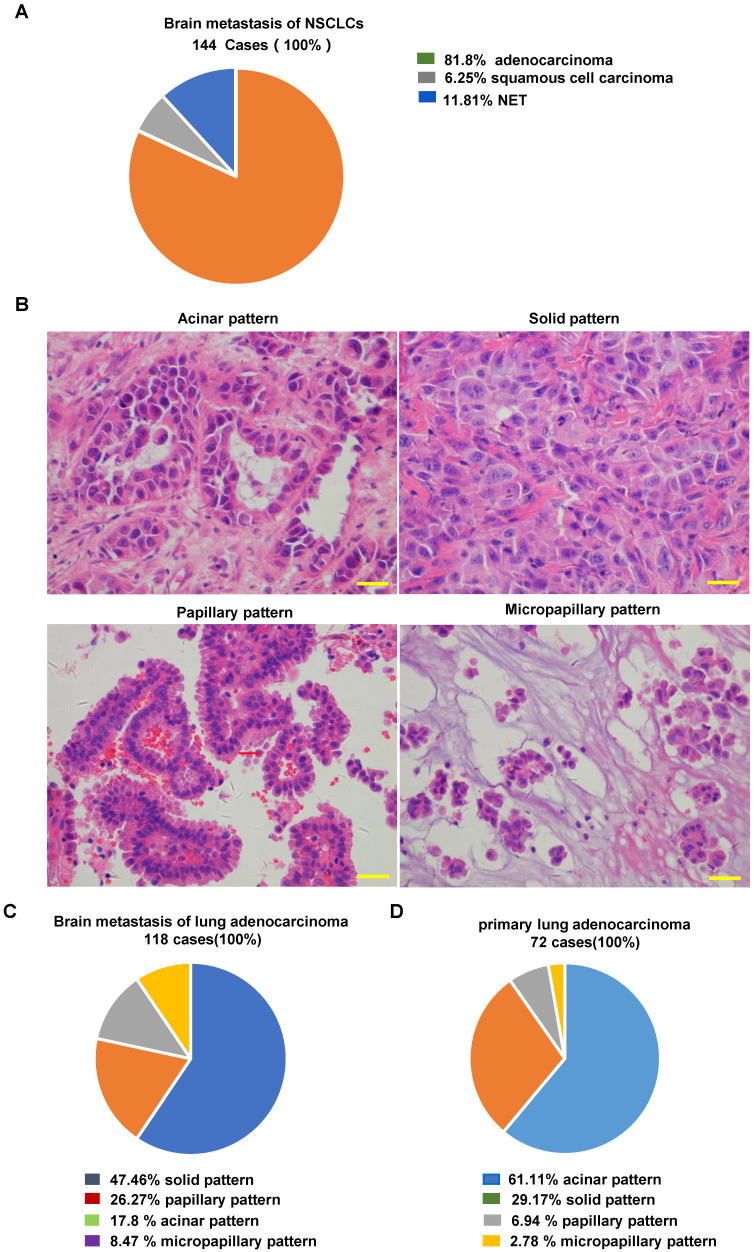
** Histopathological evaluation of NSCLC and NSCLC brain metastases. (A)** Frequency of pathologic types of 144 NSCLC brain metastases. **(B)** Representative images of the architectural pattern of brain metastatic lung adenocarcinoma (H&E). Scale bar, 50 μm. **(C)** Frequency of pathologic patterns of 118 brain metastases from lung adenocarcinoma. **(D)** Frequency of pathologic patterns of 72 primary lung adenocarcinomas. H&E: Hematoxylin and eosin.

For the 144 cases of NSCLC brain metastasis, the mean age of the patients at the time of surgical resection was 56.17 ± 10.49 years, with 80 (55.6%) males and 64(44.3%) females. Brain metastasis was infratentorial in 33 patients (22.9%), supratentorial in 110 patients (76.4%), and both supratentorial and infratentorial in 1 patient (0.7%). One hundred seventeen patients (81.2%) had a single metastasis at the time of surgical resection, and twenty-seven patients (18.8%) had multiple metastases (Supplementary Table 2S). After H&E staining, 110 of the 144 NSCLC cases showed a typical adenocarcinoma morphology. Among the remaining 34 poorly differentiated cases, we confirmed 8 cases of poorly differentiated adenocarcinoma, 9 cases of squamous cell carcinoma, and 17 cases of neuroendocrine tumors based on the necessary immunolabeling (representative images are shown in Supplementary Fig. 2S). Finally, 118 samples were identified as lung adenocarcinoma brain metastases (118/144, 81.94%) (Fig. 2A).

Representative images are shown in Fig. 2B. Among 118 lung adenocarcinoma brain metastases, the most common histopathological pattern was solid (56 cases, 47.46%), followed by papillary (31 cases, 26.27%), acinar (21 cases, 17.8%), and micropapillary (10 cases, 8.47%) (Fig. 2C). In contrast, the most common histopathological pattern in primary lung adenocarcinoma was the acinar type (Fig. 2D). Our findings are consistent with the 97 previously described cases of resected brain metastases from lung adenocarcinoma 19. These findings indicate that adenocarcinoma with a solid growth pattern has a higher propensity for brain metastasis.

### BCAT1 expression was elevated in NSCLC brain metastases

**Figure 3:**
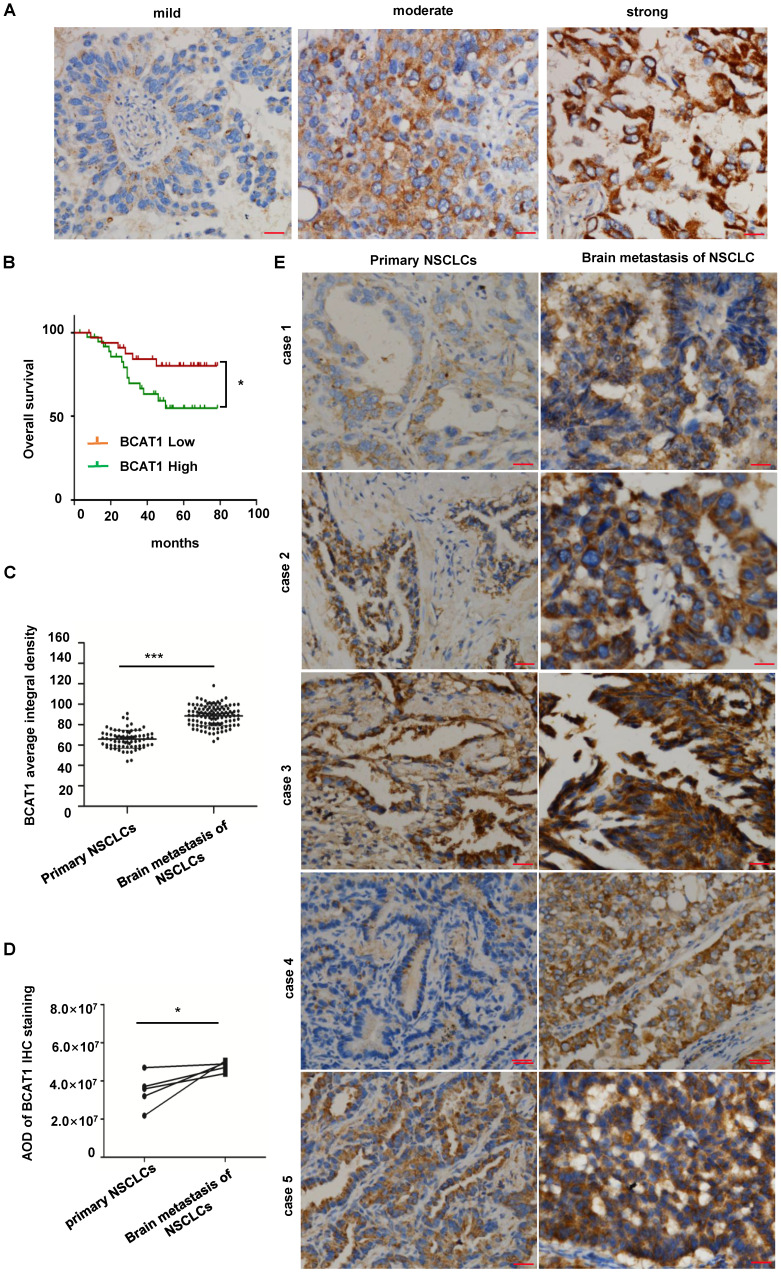
** BCAT1 was significantly overexpressed in NSCLC brain metastases. (A)** Representative images of BCAT1 immunohistochemical staining in NSCLC. **(B)** Survival curves of the BCAT1 low expression group and high expression group in primary lung NSCLC (*P = 0.035). **(C)** Statistical analysis of BCAT1 immunohistochemical staining intensity in 72 cases of primary NSCLC and 114 cases of brain metastasis of NSCLC samples (independent sample t-test, *P = 0.021). **(D)** Statistical analysis of BCAT1 immunohistochemical staining intensity in 5 paired primary and brain metastasis NSCLC samples (paired t-test, * P = 0.046). **(E)** Representative images of BCAT1 immunohistochemical staining in 5 paired primary and brain metastasis NSCLC samples. All data are shown as the mean ± SEM. Significance was assessed with Student's independent and paired t-tests. *P < 0.05. **P < 0.01, *** P < 0.001. Scale bar, 50 μm.

BCAT1 is the major BCAT isotype in NSCLC. Based on the analysis of BCAT1 immunostaining (Fig. 3A), we subdivided 72 lung primary adenocarcinoma samples into a high BCAT1 expression group (including 38 cases) and a low expression group (including 34 cases). The overall survival time (OS) for patients in the high BCAT1 expression group (56.18 ± 6.02 months) was significantly lower than that in the low expression group (69.99 ± 6.91 months) (P = 0.035) (Fig. 3B). These data indicate that patients with high BCAT1 protein level in lung adenocarcinoma tissues were associated with a poor prognosis.

We then compared BCAT1 expression in 72 lung adenocarcinoma samples and 118 lung adenocarcinoma brain metastases. BCAT1 expression was significantly higher in lung adenocarcinoma brain metastases than in primary lung adenocarcinoma (P = 0.021) (Fig. 3C). In five paired primary and brain metastatic lung adenocarcinoma samples, BCAT1 expression was also higher in brain metastatic lesions than in primary lung lesions, P = 0.046 (Fig. 3D). Representative images are shown in Fig. 3E. These data indicate that the metabolism of BCAAs is active in NSCLC brain metastases.

### BCAT1 regulates EMT transformation in NSCLC cells

**Figure 4:**
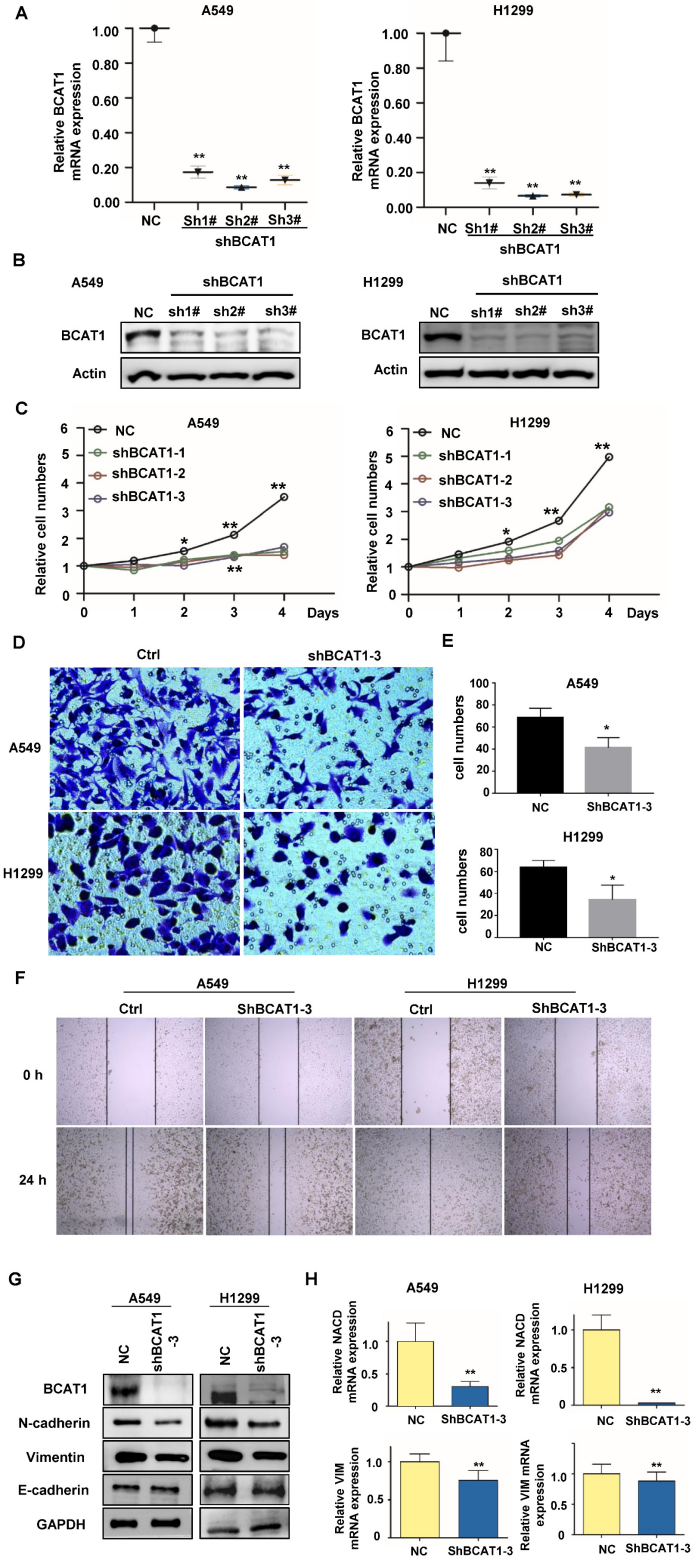
** Loss of BCAT1 leads to inhibition of EMT in NSCLC cells. (A)** The mRNA level of BCAT1 was inhibited by shRNA (sh-BCAT1) in A549 and H1299 cells. Empty vector-transfected cells were used as a normal control (NC). **(B)** The expression of BCAT1 was knocked down by shRNA. GAPDH was used as a loading control. **(C)** CCK-8 assays indicate the viability of A549 and H1299 cells in the sh-BCAT1 and Vec groups. **(D, E)** Representative images and statistical analysis of Transwell assays of A549 and H1299 cells in the sh-BCAT1 and Vec groups.** (F)** Representative images of wound healing assays of A549 and H1299 cells in the sh-BCAT1 and NC groups at 0 h and 24 h.** (G)** Western blot analysis verified that the expression of N-cadherin and vimentin in NSCLC cells significantly decreased after BCAT1 knockdown. **(H)** qRT‒PCR confirmed that the mRNA expression of N-cadherin and vimentin in lung adenocarcinoma cells decreased after BCAT1 knockdown. All data are shown as the mean ± SEM. The results are representative of three independent experiments. Significance was assessed by Student's t-tests and one-way analysis of variance. *P<0.05. **P<0.01. CCK-8: Cell Counting Kit-8.

To verify the functions of activated BCAA metabolism in NSCLC brain metastases, we knocked down BCAT1 using shRNA in A549 and H1229 cells, and verified by qRT-PCR and western-blot (Fig. 4A, B). We found that BCAT1 knockdown suppressed the growth of NSCLC cells from Day 2 after shRNA transfection (Fig. 4C). The capacities of NSCLC cells to invade (Fig. 4D, E) and migrate (Fig. 4F) were also diminished after BCAT1 knockdown.

Distant metastasis is one of the most serious pathological events in malignancy. Tumor cells that metastasize acquire the ability to separate from the tumor bulk, spread into the circulation, and extravasate to favorable metastatic sites, such as the bone, brain, and liver. In this complex process, tumor cells undergo a cellular process known as epithelial-mesenchymal transition (EMT). We subsequently examined markers associated with EMT. Compared to BCAT1 wild-type cells, Vimentin and N-cadherin (both as mesenchymal markers) were significantly reduced after BCAT1 knockdown, and the expression level of E-cadherin (the epithelial marker) is the same as those in the vehicle control group (Fig. 4G).

Similarly, the expressions of mRNAs of N-cadherin (encoded by NCAD) and vimentin were also decreased after BCAT1 knockdown (Fig 4H). The occurrence of EMT is orchestrated by several transcription factors. We found that the mRNAs of three transcription factors, ZEB1, Snail, and Slug, were unchanged after BCAT1 silencing. The mRNA levels of E-cadherin (encoded by ECAD) remained unchanged (Supplementary Fig. S3).

These data suggest that BCAT1-induced EMT may be independent of transcription factors. The expression of BCAT1 triggers the EMT process by regulating the expression of mesenchymal proteins at the transcriptional level.

### ALKBH5 regulates the expression of EMT-related mRNA

**Figure 5:**
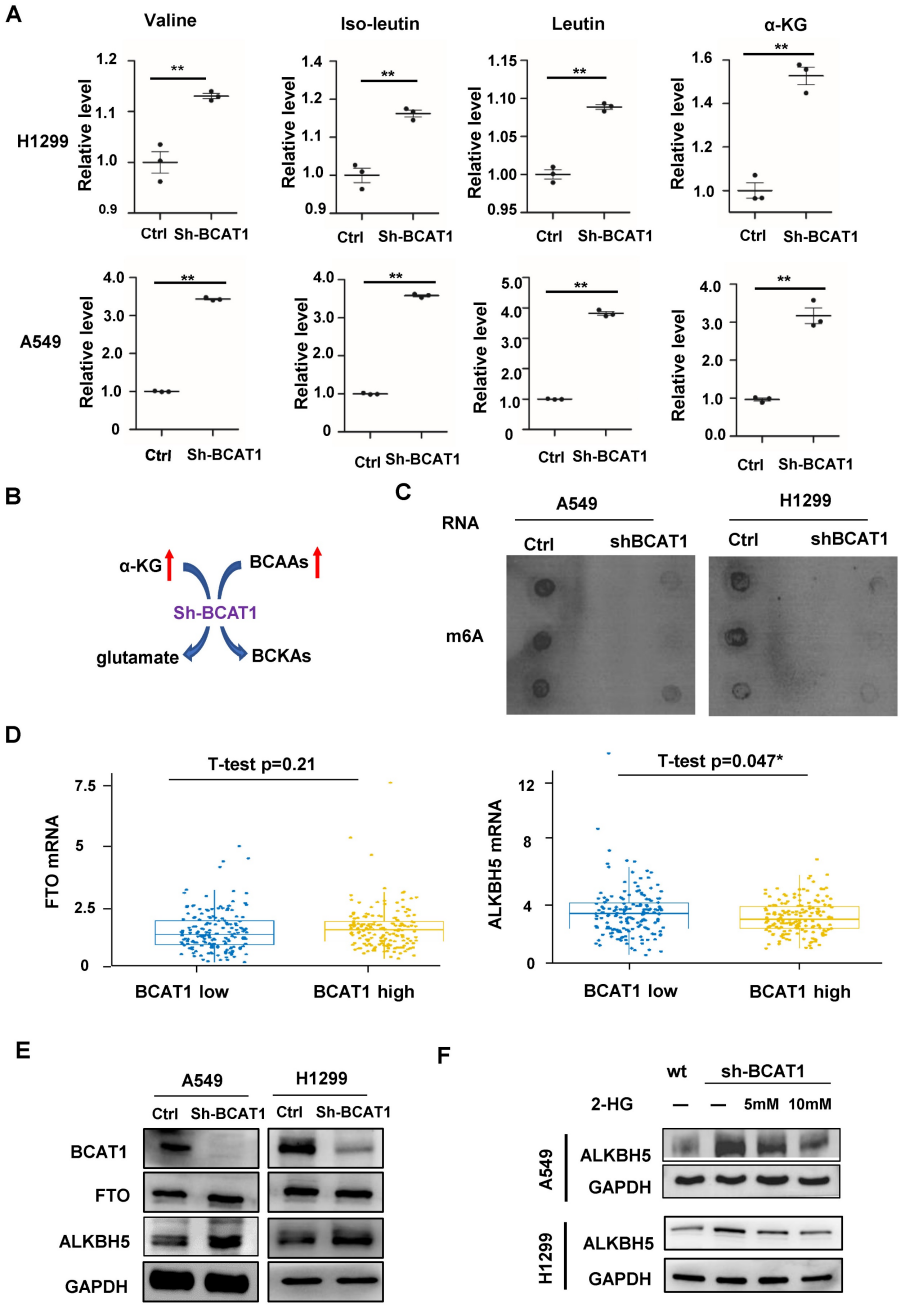
** Loss of BCAT1 leads to metabolic reprogramming and impairs the expression of ALKBH5. (A)** Quantification of the abundance of BCAAs and α-KG in NSCLC cells with or without BCAT1 knockdown via LC-MS/MS analysis. **(B)** Schematic diagram of BCAA catabolism by BCAT1 in NSCLC cells. **(C)** In A549 and H1299 cells, the m6A levels in mRNA decreased after BCAT1 knockdown. **(D)** In NSCLC samples from TCGA data, BCAT1 expression was positively correlated with the level of ALKBH5 (* P=0.047), while no relationship was observed between the expression of BCAT1 and FTO (P = 0.48). **(E)** BCAT1 knockdown did not change FTO expression, whereas ALKBH5 expression was increased in NSCLC cells. **(F)** Following treatment with the α-KG analog 2-HG, the BCAT1 knockdown-induced increase in AKLBH5 expression was rescued in NSCLC cells. GAPDH was used as a loading control in all immunoblots. All data are shown as the mean ± SEM. The results are representative of three independent experiments. Significance was assessed by Student's t-tests. *P<0.05. **P<0.01.

BCAT1 is a highly active and reversible enzyme that acts on all three BCAAs and their corresponding BCKAs. Thus, BCAT1 converts BCAAs into their corresponding branched-chain ɑ-keto acids by transferring an amino group to α-KG to generate glutamate. BCAT can also transfer nitrogen from glutamate back to BCKAs and regenerate BCAAs and α-KG. Thus, cells rapidly exchange nitrogen between BCAAs, BCKAs, glutamate, and α-KG. Therefore, after inhibition of BCAT1 expression in lung adenocarcinoma cells, we first examined the levels of BCAAs and α-KG in cells via LC-MS/MS. We found that inhibition of BCAT1 in lung adenocarcinoma cells increased the cellular contents of the three BCAAs and α-KG (Fig. 5A), which is illustrated by the schematic in Fig. 5B. In mammalian cells, there are more than 60 dioxygenases that utilize α-KG as a cosubstrate 20, 21. As a family of α-KG-dependent dioxygenases, ten-eleven translocation (TET) proteins catalyze the sequential oxidation reaction required for DNA demethylation that converts 5mC to 5-hydroxymethylcytosine (5hmC) 22. We did not find any changes in 5hmC levels after BCAT1 knockdown (Supplementary Fig. S4**)**. N6-methyladenine (m6A) is a dynamic and reversible mRNA modification, the most abundant internal mRNA modification, and is extensively involved in metabolism, affecting mRNA stability. To date, two eukaryotic m6A demethylases, also known as m6A erasers, have been identified: fat mass and obesity-associated protein (FTO) and AlkB homolog 5 (ALKHB5). Both are α-KG-dependent dioxygenases 23. We found that m6A modifications were reduced after BCAT1 knockdown in NSCLC cells (Fig. 5C). In NSCLC samples from TCGA, the expression of ALKBH5 but not FTO was negatively correlated with BCAT1 levels (Fig. 5D). We verified that the expression of ALKBH5, but not FTO, was increased after BCAT1 knockdown in NSCLC cells (Fig. 5E). Replenishment of 2-HG, a well-known α-KG competitor, reversed the increase in ALKBH5 in NSCLC cells induced by BCAT1 knockdown (Fig. 5F). All these data suggest that the upregulation of ALKBH5 after BCAT1 inhibition is associated with α-KG.

### ALKBH5 regulates the expression of EMT markers in NSCLC

**Figure 6:**
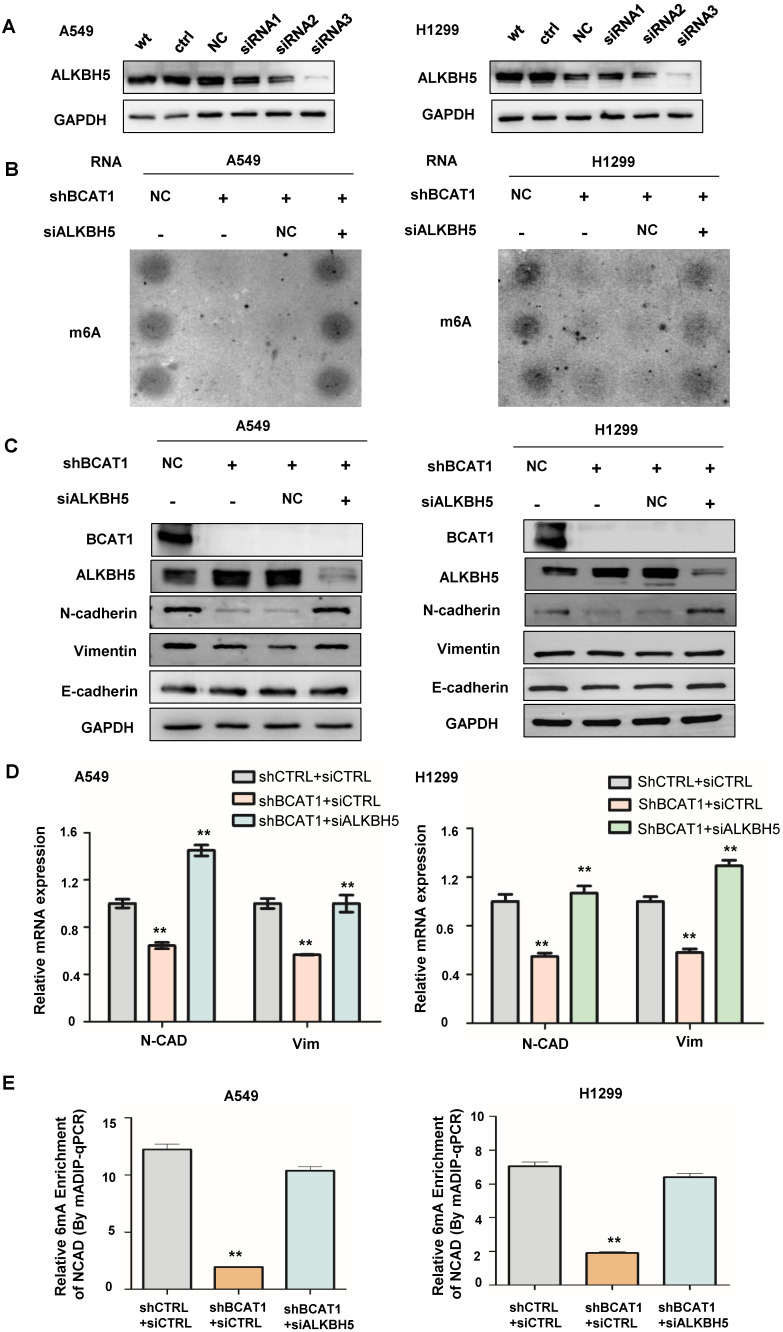
** ALKBH5 mediated the effects of BCAT1 on mesenchymal gene expression in NSCLC cells. (A)** Reduction of ALKBH5 expression in NSCLC cells by siRNA. **(B)** Disruption of m6A modification of mRNA by BCAT1 knockdown in NSCLC cells was rescued by ALKBH5 inhibition. **(C)** ALKBH5 inhibition rescued the levels of N-cadherin and vimentin induced by BCAT1 knockdown. GAPDH was used as a loading control in all immunoblots. **(D)** Inhibition of ALKBH5 rescued the expression of VIM and NCAD mRNA induced by BCAT1 knockdown. **(E)** Inhibition of ALKBH5 rescued the decrease in NCAD m6A methylation after BCAT1 knockdown in NSCLC cells, as shown by MeRIP-PCR. All data are shown as the mean ± SEM. The results are representative of three independent experiments. Significance was assessed by one-way ANOVA with a post hoc test (e.g., Dunnett's test). *P<0.05. **P<0.01.

Since ALKBH5 is an α-KG-dependent m6A demethylase, we wondered about its role in the BCAT1-induced EMT process. We found that the knockdown of ALKBH5 by siRNA (verified by western blotting, shown in Fig. 6A) rescued the m6A modification inhibited by BCAT1 knockdown in NSCLC cells (Fig. 6B). Consistent with this result, the knockdown of ALKBH5 in NSCLC cells with BCAT1 knockdown rescued the expression of N-cadherin and vimentin (Fig. 6C-D). Using MeRIP-PCR, we also confirmed that the levels of m6A methylation of NCAD were decreased after BCAT1 knockdown in NSCLC cells and rescued after ALKBH5 inhibition (Fig. 6E).

### BCAT1 inhibition impedes the proliferation of NSCLC cells in the brain

**Figure 7:**
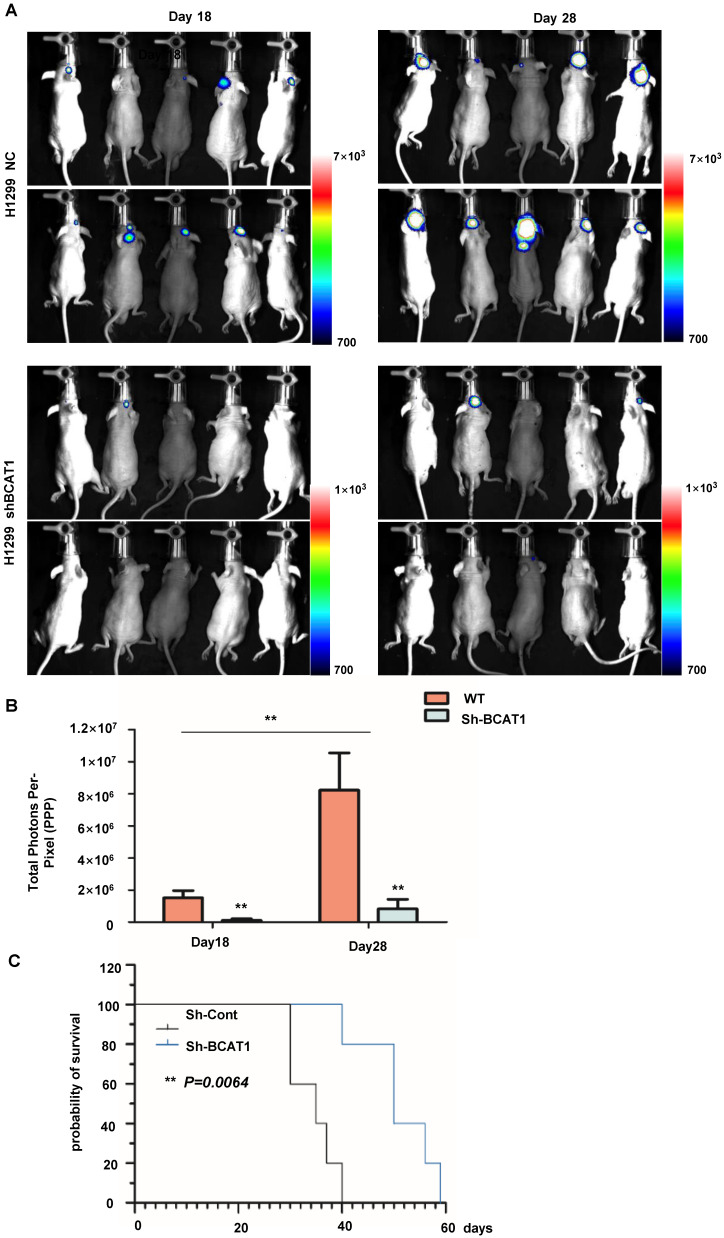
** BCAA knockdown reduced the NSCLC cell burden in the brain. (A)** Representative *in vivo* pseudocoloured bioluminescence images of nude mice transplanted with control or BCAT1-KD NSCLC cells. **(B)** BCAT1 knockdown reduced the NSCLC burden *in vivo*. The unit of radiance is “photons/second/cm^2^/steradian”. All data are shown as the mean ± SEM. Significance was assessed by Student's t-tests. **(C)** Survival time of H1299 cells in a stereotactically injected nude mouse model. Mice in the BCAT1 knockout group survived significantly longer than mice in the BCAT1 wild-type group. Each group contained 10 biologically independent mice (**P = 0.00064).

We used a tumor stereotactic injection mouse model to examine the effect of BCAA catabolism on the proliferation of NSCLC cells in the brain. The luciferase activity of intracranially injected H1299 cells was detectable within 20 days after implantation, with a marked and steady increase in tumor luminescence by Day 28 post-implantation. As expected, BCAT1 knockdown cells exhibited less robust luciferase activity than control cells (Fig. 7A, B). The median overall survival times (OS) of nude mice transplanted with BCAT1 wild-type H1299 cells and BACT1-deficient H1299 cells producing tumors were 35.0 and 50.0 days, respectively, indicating a significant difference in OS time between the two groups (P = 0.0064) (Fig. 7C).

**Figure 8:**
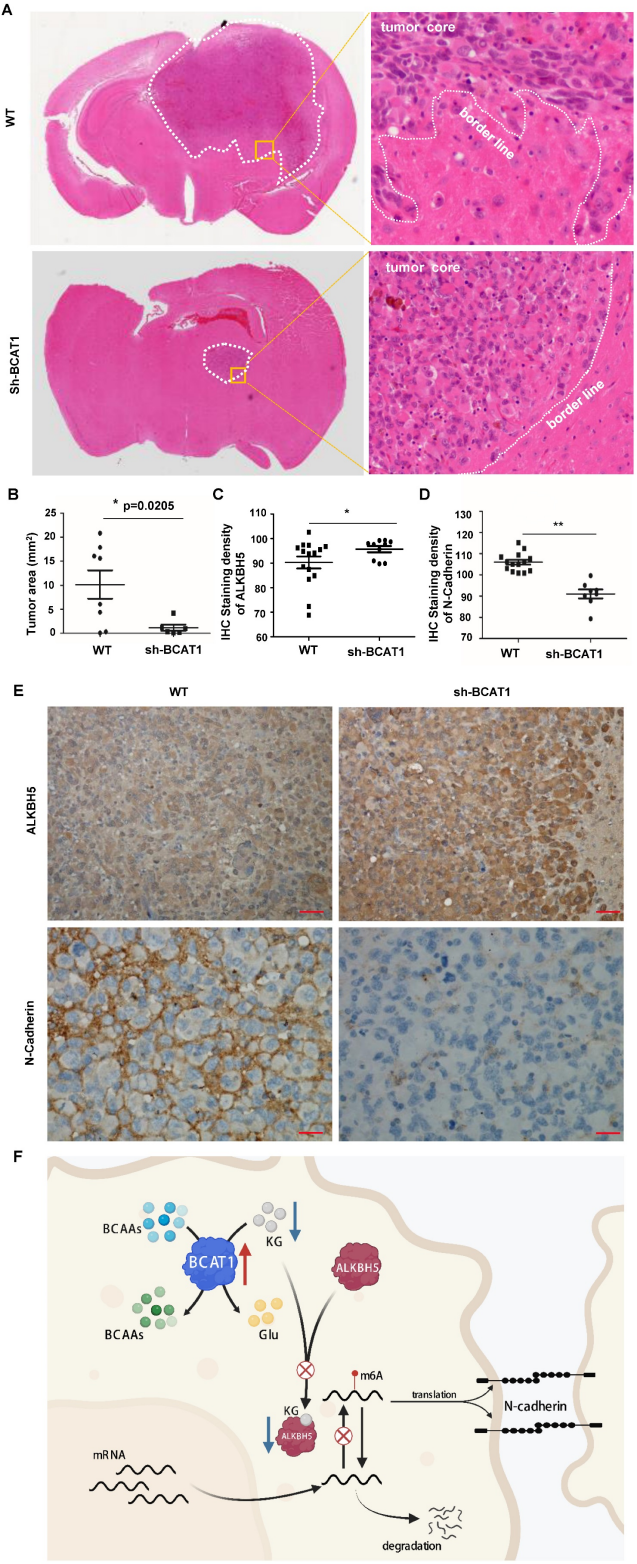
** BCAAs sustain the invasive growth of NSCLC cells in the brain. (A)** Representative H&E staining images of stereotactically transplanted NSCLC cells. **(B)** Quantification of brain regions affected by NSCLC cells. Tumor volume was significantly reduced after BCAT1 knockdown. Mean±SEM, two-tailed t-test, P=0.0205. **(C)** Statistical analysis of ALKBH5 IHC integral optical density. The expression of ALKBH5 in the BCAT1-KD group was significantly higher than that in control samples (P = 0.02). **(D)** Statistical analysis of N-cadherin IHC integral optical density. The expression of N-cadherin was significantly lower in the BCAT1-KD group than in the control samples (P = 0.02). **(E)** Representative images of IHC staining of ALKBH5 and N-cadherin in orthotopically transplanted tumors. All data are shown as the mean ± SEM. Significance was assessed by Student's t-test. Scale bar, 50 μm. **(F)** Proposed molecular mechanism model of BCAA catabolism participation in EMT of NSCLC cells.

In the BCAT1 wild-type group, the tumor cells infiltrated into the surrounding brain tissues, whereas in the BCAT1 knockdown group, the tumors had distinct and clear borders (representative images are shown in Fig. 8A). The BCAT1 knockdown group had a significantly reduced growth area of NSCLC cells compared with the control group (Fig. 8B). These findings indicated that the proliferation of BCAT1-deficient cells in the brain was attenuated. Through IHC staining, we found that the expression of ALKBH5 was increased in the BCAT1 knockdown group compared to the wild-type group (P = 0.0429) (Fig. 8C). The expression level of N-cadherin, a marker of mesenchymal status, was decreased in the shBCAT1 groups compared to the wild-type group (P = 0.0002) (Fig. 8D). Representative images of IHC staining of ALKBH5 and N-cadherin are shown in Fig. 8E. These findings are consistent with those found in NSCLC cells cultured *in vitro*.

## Discussion

An increasing number of studies have focused on the metabolism of BCAAs in tumor cells. In tumors, such as ovarian epithelial carcinoma, gastric cancer, breast cancer, and lung cancer, BCAAT1 expression is elevated and is closely associated with the aggressive growth of tumor cells 24-27. However, the mechanism by which BCAA metabolism is upregulated to support the aggressive growth and distant metastasis of tumor cells remains unclear. For BCAA metabolism, BCAT is the enzyme responsible for the reversible transamination of BCAAs, producing corresponding BCKAs. The latter is further decarboxylated to produce acetyl-CoA and succinyl-CoA, which enter the mitochondrial tricarboxylic acid cycle and provide energy for ATP synthesis and the synthesis of lipids and nucleotides 28. In addition, the consumption of α-KG is necessary for the production of glutamate. The dioxygenases that utilize α-KG as a substrate, including histone demethylases, DNA demethylases, and mRNA demethylases, are involved in various cell activities 21.

The m6A methylation modification is the most common mRNA epigenetic modification and requires the joint participation of methyltransferases, demethylases, and methylated reading proteins 29, 30. The demethylases known to catalyze mRNA demethylation are FTO and ALKB5, which both belong to the ALKB family of Fe^2+^/α-KG-dependent oxygenases but exhibit different substrate preferences. FTO has a broader substrate preference, including m6A, hm6A, N1 methyladenosine (m1A), and N6, 2'-O-dimethyladenosine (m6Am), while ALKBH5 is more specific for m6A modification of mRNA 23. Recently, Jin et al. showed that ALKBH5 plays an essential role in the invasive growth of lung cancer cells 31. Our analysis of TCGA data showed that BCAT1 expression was not correlated with FTO expression in NSCLC but was negatively correlated with ALKBH5 expression. We found that the expression of ALKBH5 increased in BCAT1 knockdown NSCLC cells without a change in FTO expression. We treated NSCLC cells with the α-KG analog 2-HG, which reversed the increase in ALKBH5 expression after BCAT1 knockdown. Our results suggested that the reduction in α-KG during the catabolism of BCAAs in NSCLC cells downregulates the expression and activity of the m6A demethylase ALKBH5, which would facilitate the maintenance of m6A methylation of mRNA to maintain mRNA stability and promote mRNA translation and protein expression 29.

Epithelial-mesenchymal transition (EMT) occurs in the process of invasive growth and distant metastasis of epithelial-derived malignant tumors. In the EMT process, epithelial cells gradually lose cell polarity, intercellular adhesion capacity, and tight junctions and transform into cells with interstitial cell morphology and interstitial cell characteristics, eventually acquiring migration and invasion capabilities. EMT is manifested by the loss of epithelial-type indicator proteins, such as catenin and E-cadherin, and the acquisition of interstitial-type indicator proteins, such as N-cadherin and vimentin 23. In HCC patients, the level of BCAT1 was found to be higher in circulating tumor cells. Downregulation of BCAT1 in HCC cells induced increased E-cadherin expression and decreased vimentin expression 32, suggesting an association between BCAA metabolism and EMT. We found that downregulation of BCAT reduced the mRNA and protein levels of N-cadherin and vimentin in NSCLC cells, while deletion of ALKBH5 rescued their expression. These data indicated that catabolism of BCAAs leads to changes in intracellular α-KG levels, which further regulate the malignant proliferation of NSCLC cells through epigenetic regulation of gene expression via ALKBH5 modification.

In summary, the study profiled serum metabolites of brain metastatic NSCLC patients before and after treatment, finding a significantly enriched BCAAs metabolism pathway after treatment. Adenocarcinoma was found to be the most common pathological type of NSCLC brain metastasis, with solid and micropapillary growth patterns being prevalent. The expression of BCAT1 was significantly higher in NSCLC brain metastases than in lung NSCLC. Metabolic reprogramming of BCAAs is closely associated with increased proliferation capacity of various tumor cells. The level of α-KG was significantly increased after the abolishment of BCAAs catabolism, which modulated the expression and activity of ALKBH5, an m6A demethylase involved in aggressive proliferation of NSCLC cells in the brain. Targeting BCAAs metabolism may provide a new treatment strategy for brain metastasis of NSCLC.

## Supplementary Material

Supplementary figures.

## Abbreviations

BCAAs: branched-chain amino acids
BCAT1: Branched-chain amino acid aminotransferase 1
NSCLC: Non-small cell lung cancer
EMT: epithelial-mesenchymal transition
FTO: fat mass and obesity-associated protein
ALKHB5: AlkB homolog 5
α-KG: α-ketoglutarate
LC-MS: liquid chromatography-mass spectrometry
qPCR: quantitative real-time PCR
MeRIP- PCR: m6A immunoprecipitation qPCR
DAB: 3,3-diaminobenzidine
RIPA: radioimmunoprecipitation buffer
HE: hematoxylin & eosin
