# Supplementary material for: Branch Chain Amino Acid Metabolism Promotes Brain Metastasis of NSCLC through EMT Occurrence by Regulating ALKBH5 activity
Source: Int J Biol Sci. 2024 Jun 29;20(9):3285–301. doi: 10.7150/ijbs.85672 (PMC11234221; doi:10.7150/ijbs.85672)
Supplement: Supplementary file 1 — Supplementary figures. [file ijbsv20p3285s1.pdf]

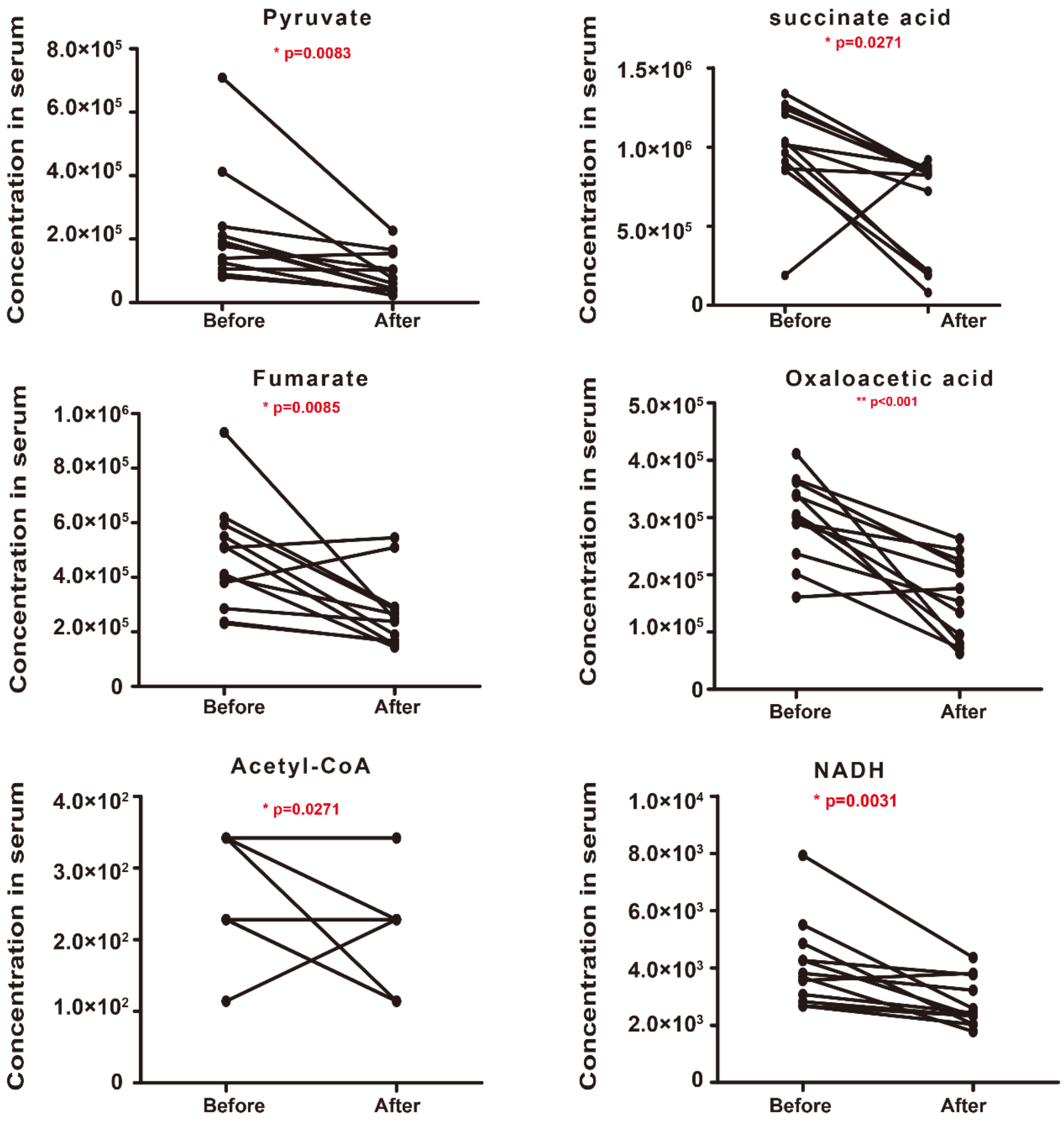

Supplementary Figure 1S. The metabolism profile indicated the decreasing of metabolites in NSCLC brain metastasis patients' blood serum after CyberKnife Stereotactic Radiotherapy. Significance was assessed with paired t-tests. *P<0.05. **P<0.01, *** P<0.001.

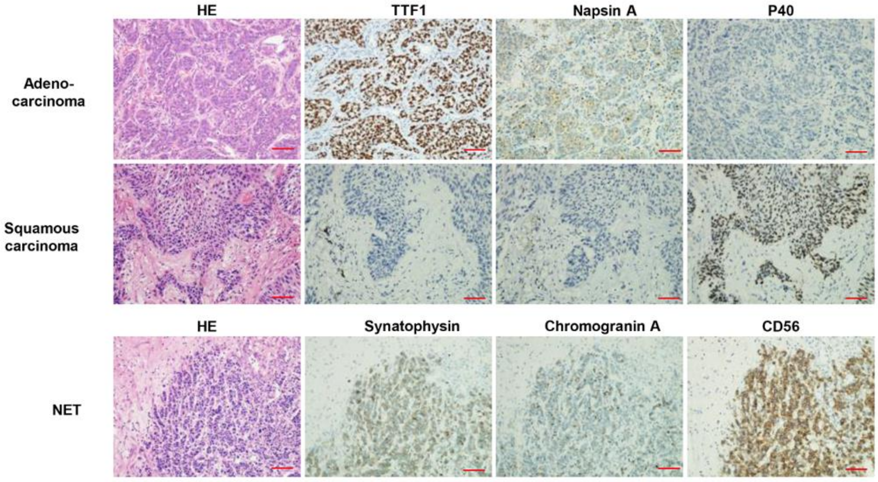

Supplementary Figure 2S. Presentative images of HE and IHC staining to differentiate adenocarcinoma, squamous carcinoma, and neuroendocrine tumor of 144 brain metastasis of lung cancer.

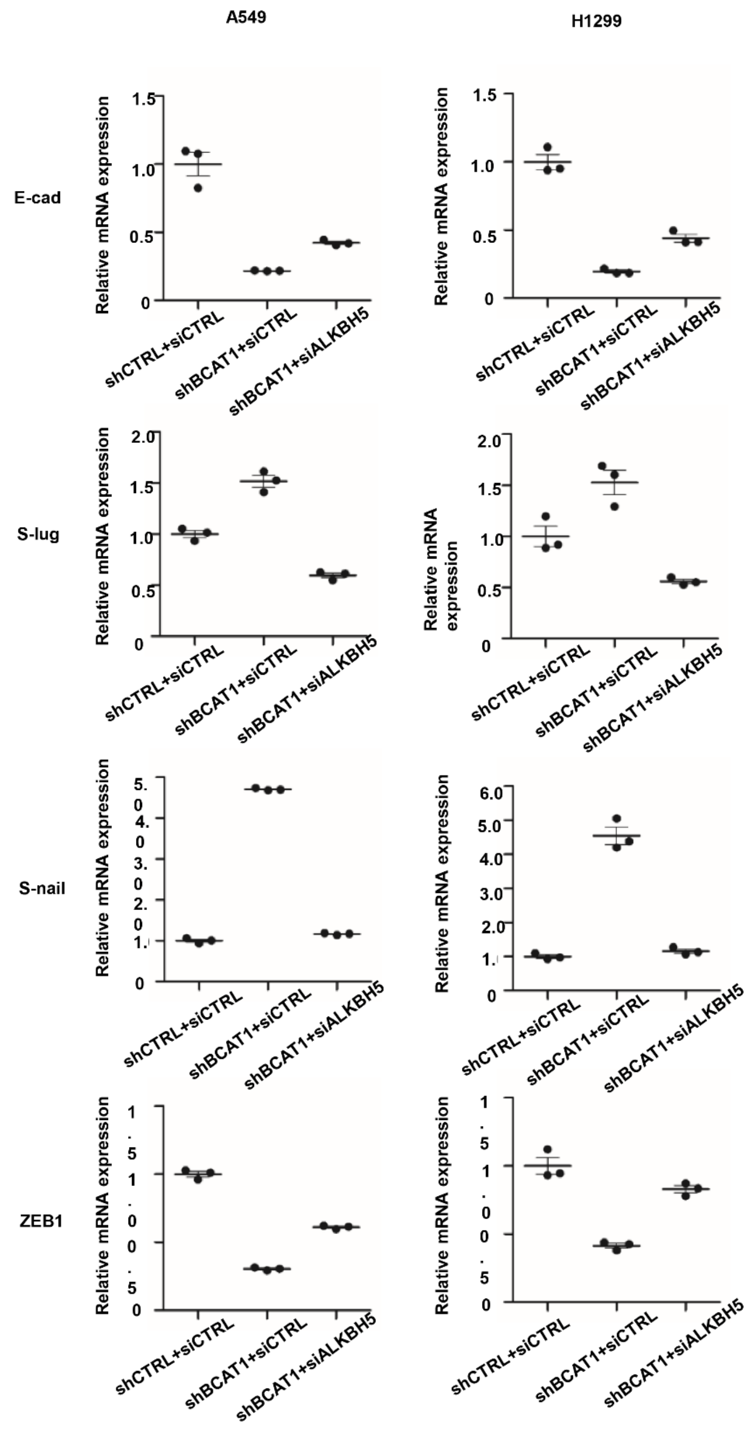

Supplementary Figure 3S. The expressions of ZEB1, SNAIL, and SLUG mRNAs after BCAT1 and ALKBH5 knockdown in NSCLC cells.

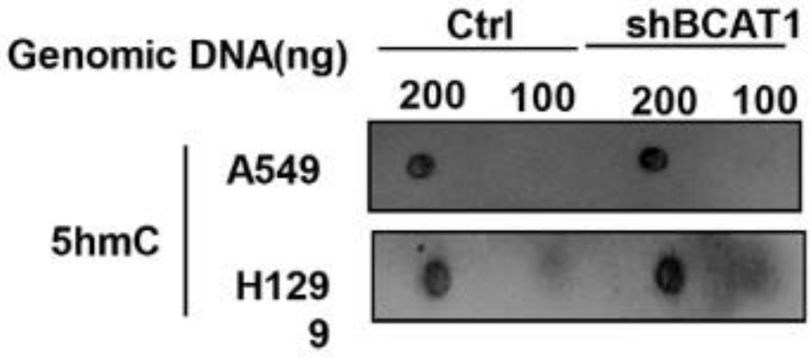

Supplementary Figure 4S. After BCAT1 knockdown, DNA demethylation of NSCLC cells showed no changes.
